# Refined identification of neutralization-resistant CRF02_AG viruses and their sensitivity to anti-MPER neutralizing antibodies

**DOI:** 10.1186/1742-4690-9-S2-P77

**Published:** 2012-09-13

**Authors:** RA Jacob, F Abrahams, M Tongo, M Schomaker, P Roux, E Mpoudi Ngole, WA Burgers, JR Dorfman

**Affiliations:** 1 International Centre for Genetic Engineering and Biotechnology, Cape Town, South Africa; 2 Division of Medical Virology, University of Cape Town, Cape Town, South Africa; 3 Centre for Infectious Disease Epidemiology & Research, UCT, Cape Town, South Africa; 4 School of Child and Adolescent Health, University of Cape Town, Cape Town, South Africa; 5 Centre de Recherche sur les Maladies Émergentes et Réémergentes, Yaoundé, Cameroon

## Background

The first antibody-inducing HIV-1 vaccines are unlikely to protect against all HIV-1 isolates. There is thus a danger that a vaccine will select for HIV-1 viruses that are highly resistant to antibody-mediated neutralization. We sought to identify and characterize such viruses.

## Methods

A diverse panel of 24 HIV-1 pseudoviruses was tested for neutralization resistance using two sets of samples from ARV-naive HIV-1-infected individuals selected for good neutralizers: sera from South Africa donors (n=68, infected >1 year, subtype C predominant area) and CRF02_AG-infected plasma samples from Cameroon donors (n=12, good neutralizers selected from 22 samples).

## Results

Sensitivity to South Africa sera by subtype was C>B≈CRF02_AG>A. Importantly, and in contrast to previous reports, CRF02_AG plasma neutralized CRF02_AG viruses better than other panel viruses (“within-subtype neutralization”). This included three (257-31, 251-18 and 33-7) of five CRF02_AG viruses previously designated as tier 3 (most resistant). This within-subtype neutralization testing showed that the other two tier 3 CRF02_AG panel viruses, 253-11 and 278-50 were highly resistant. Most CRF02_AG viruses, including 253-11 and 278-50 were sensitive to two membrane proximal external region (MPER)-specific monoclonal antibodies and soluble CD4 (sCD4), suggesting targets for neutralization of even these highly resistant viruses. This information may help design a global HIV-1 vaccine. We also propose testing viruses with within-subtype samples selected for good neutralizers in order to evaluate their neutralization resistance.

## Conclusion

Some but not all CRF02_AG viruses are sensitive to neutralisation by CRF02_AG-derived plasma, even though most are previously reported as highly resistant (Tier 3). Further work is necessary to properly characterize such Tier 3 viruses. If research focus is not placed on such resistant viruses, a future partially effective HIV-1 vaccine may select for them.

